# Cardiovascular Effects of Androgen Deprivation Therapy in Prostate Cancer Patients: A Systematic Review

**DOI:** 10.7759/cureus.26209

**Published:** 2022-06-22

**Authors:** Meghana Kakarla, Musa Ausaja Gambo, Mustafa Yousri Salama, Nathalie Haidar Ismail, Pardis Tavalla, Pulkita Uppal, Shaza A Mohammed, Shriya Rajashekar, Suganya Giri Ravindran, Pousette Hamid

**Affiliations:** 1 Internal Medicine, California Institute of Behavioral Neurosciences & Psychology, Fairfield, USA; 2 Pediatrics and Child Health, California Institute of Behavioral Neurosciences & Psychology, Fairfield, USA; 3 Medicine, California Institute of Behavioral Neurosciences & Psychology, Fairfield, USA; 4 Neurology, California Institute of Behavioral Neurosciences & Psychology, Fairfield, USA

**Keywords:** cardiovascular disease, cardiotoxicity, gonadotropin-releasing hormone (gnrh) agonist, prostate cancer (pca), anti-androgen therapy, surgical castration, orchiectomy, prostate neoplasm, adverse cardiovascular events, androgen deprivation therapy

## Abstract

The purpose of this study was to investigate the relationship between androgen deprivation therapy (ADT) and cardiovascular events in men with prostate cancer. Cardiovascular disease (CVD) is a primary cause of noncancer mortality in men with prostate cancer. Surveillance, Epidemiology, and End Results (SEER) Medicare-linked data revealed that CVD was responsible for about a quarter of deaths among men with prostate cancer, with a focus on the role of ADT as a contributing cause.

We performed a literature search in November 2021 utilizing search engines such as PubMed, Scopus, Science Direct, and Google Scholar. Original publications with data published between 2006 and 2020 were used in the investigation of men with prostate cancer undergoing ADT treatment with a CVD outcome. Two reviewers independently examined the content of the studies and extracted data from the final papers after they had been validated for quality using quality assessment tools. A total of 14 observational studies and two randomized controlled trials are included in this systematic review. Sample sizes in the examined publications varied from 79 to 201,797 individuals. ADT was the intervention in all of the investigations. Seven of the included studies did not identify the type of ADT utilized; instead, they compared the outcomes of individuals who got ADT against those who did not. The specific type of ADT used is mentioned in the remaining nine studies included in the systematic review. Patients who got ADT, such as gonadotropin-releasing hormone (GnRH) agonists, combination androgen blockade, surgical castration, and oral anti-androgen, are compared to those who did not receive ADT to discover who had a better prognosis.

In conclusion, even though ADT has several negative metabolic side effects that increase the risk of cardiovascular toxicity, published research utilizing a variety of designs has demonstrated inconsistency in the impact of ADT on cardiovascular outcomes. While the risk of CVD should be considered when prescribing ADT, the findings suggest that it should not be considered a contraindication if the expected benefit is substantial.

## Introduction and background

In developed countries where there are large populations of older men, prostate cancer becomes a serious public health problem as its prevalence increases throughout life. In the United States, following lung cancer, prostate cancer is the second-highest cause of cancer death in males, with one estimate claiming that 1.3 million newly diagnosed cases of prostate cancer were recorded in men in 2018 associated with 359,038 deaths [1].

**Figure 1:**
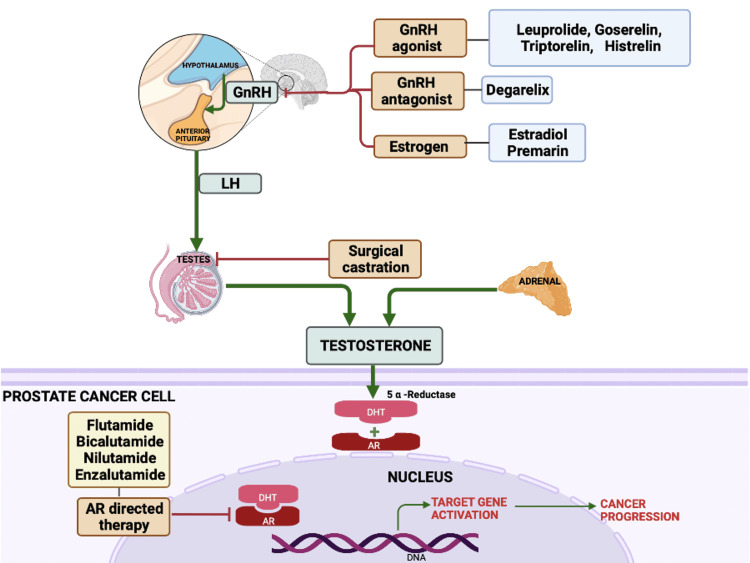
The hypothalamic-pituitary-gonadal axis and sites of action of androgen deprivation therapy. GnRH: gonadotropin-releasing hormone; LH: luteinizing hormone; DHT: dihydrotestosterone; AR: androgen receptor Created by the authors using biorender.com.

Since the first observation by Huggins and Hodges in 1941, it has been established that androgens (chiefly testosterone) play a key role in promoting tumor growth in prostate cancer patients [2]. Hence, effective anti-cancer therapy revolves around declining exposure to androgens and is referred to as androgen deprivation therapy (ADT). ADT is frequently used with localized treatments such as external beam radiotherapy or brachytherapy [3]. Even though orchiectomy (surgical castration) is a simple and cheap procedure, it is less common due to its irreversibility [4]. Multiple strategies are employed to reduce exposure to testosterone including surgically removing the testes that produce 90% of the hormone or undergoing hormonal therapy which entails either reducing testosterone secretion or blocking androgen receptors. Figure 1 depicts the hypothalamic-pituitary-gonadal axis and the sites of action of ADT.

ADT has become more common in prostate cancer patients in recent years with approximately 40% of men undergoing ADT within six months of diagnosis [5]. Cooperberg and colleagues documented the significant increase in the use of ADT from 1989 to 2001 in their report. The most dramatic change was the increase in the use of ADT in external beam radiation therapy (RT) from 9.8% to 74.6% of patients [6].

The use of ADT in prostate cancer treatment has expanded beyond symptomatic metastatic disease treatment to include asymptomatic metastatic disease, primary treatment in localized disease when men are unable to undergo surgery or radiotherapy, adjunct therapy in high-risk diseases treated with radiotherapy, and salvage therapy after relapse after surgery or radiotherapy for presumed localized disease [7,8]. The European Association of Urology (EAU) guidelines recommend ADT for patients with metastatic disease (level of evidence: A) or combined with RT for individuals with high-risk cancers (level of evidence: A). Further, ADT can be used as a single treatment for men with advanced prostate cancer who refuse, are incapable to take any other form of local treatment, or are asymptomatic, with a prostate-specific antigen (PSA) higher than 50 ng/mL and a tumor that is not well-differentiated (level of evidence: A) [9].

A profound testosterone deficiency created by ADT can cause various adverse short and long-term health effects, including hot flushes, sexual dysfunction, obesity, sarcopenia, dyslipidemia, hyperinsulinemia, osteoporosis, type 2 diabetes mellitus (DM), and cardiovascular disease (CVD) [5,7,8]. Despite the fact that male sex is a known risk factor for coronary artery disease (CAD), evidence is mounting that testosterone may protect men with prostate cancer and men in general against heart disease [8]. ADT has been shown to promote adiposity in males; one study reported that after one year of ADT, body fat increased by 9.4% [10].

Most patients with prostate cancer have CVD as comorbidity [11-13]. In men with prostate cancer, CVD is the leading cause of non-cancer mortality. In the mid-1990s, Surveillance, Epidemiology and End Result (SEER) Medicare-linked data showed that CVD was responsible for around one-fourth of deaths among men with prostate cancer [14]. This piqued the researchers’ interest in determining what might be causing CVD in this group, focusing on the role of ADT as a contributory factor. In 2010, the American Heart Association, the American Society for Radiation Oncology, and the American Urological Association issued a joint statement to raise awareness of the ADT-CVD relationship [15].

In males with prostate cancer, significant increases in total blood cholesterol and triglyceride levels have also been related to androgen deficiency [16]. CAD is exacerbated by obesity and hyperlipidemia, both of which are risk factors [16]. ADT also raises hemoglobin A1C levels in men who already have DM [17]. Although more research is needed to demonstrate that the link between CVD and ADT is not due to confounding factors, there are multiple physiologically possible mechanisms through which ADT may contribute to CVD development [17].

Dyslipidemia, DM, and obesity are all well-known possible causes of atherosclerotic CVD [18]. Androgens may influence the local inflammatory response, which plays a critical role in the formation of atherosclerotic plaques, as well as plaque instability and rupture, via androgen receptor (AR)-dependent and AR-independent pathways, according to recent studies [17]. As a result, these consequences of medically induced hypogonadism may provide a mechanism through which ADT may raise the risk of cardiac morbidity and mortality. Hence, a thorough systematic review of published papers was performed to precisely analyze the relationship of ADT with cardiovascular events in prostate cancer patients and to assist healthcare providers in making related clinical decisions [10].

## Review

Methodology

The Preferred Reporting Items for Systematic Reviews and Meta-Analyses (PRISMA) standards were used to perform this systemic review [19].

Database and Search Strategy

The research was started on November 20, 2021, using online libraries as our database. We searched PubMed, Scopus, Science Direct, and Google Scholar for data collection. Our keywords and medical subject heading (MeSH) search strategies used are depicted in Table 1.

**Table 1:** Keywords and medical subject heading search strategies used in the review.

Database	Search strategies
PubMed	“Prostatic Neoplasms/drug therapy”[Majr] OR “Prostatic Neoplasms/therapy”[Majr] AND “Antineoplastic Agents, Hormonal”[Mesh] AND “Cardiovascular Diseases”[Mesh]) AND (androgen deprivation therapy)
Science Direct	Androgen deprivation therapy OR hormonal antineoplastic therapy AND prostate neoplasm AND cardiovascular effects
Google Scholar	Cardiovascular effects of androgen deprivation therapy in prostate cancer
Scopus	Prostate AND neoplasm AND androgen AND deprivation AND therapy AND cardiovascular AND effects

Eligibility Criteria

Studies investigating the association of ADT with cardiovascular events in prostate cancer patients were included without geographic location or publication status restrictions. The following selection criteria were included: (1) the study was published as an original article and contained the original data (excluding reviews, editorials, and conference summaries); (2) only included patients diagnosed with prostate cancer; (3) the intervention group included ADT (medical or surgical ADT); (4) studies with cardiovascular outcomes as the endpoint. Studies were excluded if any of the following factors were identified: (1) secondary studies; (2) laboratory studies; (3) animal studies; (4) database duplication and lack of detailed results.

Quality Assessment Tools

The assessment was separately conducted by two independent reviewers using the Cochrane risk bias assessment tool for clinical trials and the modified version of the Newcastle-Ottawa Scale (NOS) for quality assessment of observational studies [19]. With the help of another field expert, all discrepancies regarding the inclusion of the studies were resolved. High-quality studies were defined as those that received 70% or more of the highest number of stars. We excluded studies that were of low quality. In total, 16 studies were included [4,19-33]. Table 2 shows the quality assessment of clinical trials using the Cochrane Risk of Bias Tool.

**Table 2:** Cochrane Risk of Bias Tool.

Citation	Random sequence generation	Allocation concealment	Selective reporting	Blinding (participants and personnel)	Blinding (outcome assessment)	Incomplete outcome data
D’Amico et al. [19]	L	L	L	L	L	L
Efstathiou et al. [20]	L	L	L	L	L	L
%	100	100	100	100	100	100

Table 3 shows the quality assessment of cohort studies using the modified version of the NOS.

**Table 3:** Modified version of the Newcastle-Ottawa Scale: cohort studies.

Author	Year	Selection (maximum of four stars)				Comparability (maximum of two stars)	Outcome (maximum of three stars)			Total score(Maximum of nine stars)
		Representativeness of the exposed cohort	Selection of the non-exposed cohort	Ascertainment of exposure	Demonstration that the outcome of interest was not present at the start of the study	Control for important or additional factors	Assessment of outcomes	Was follow-up long enough for outcomes to occur	Adequacy of follow-up of cohorts	
Keating et al. [23]	2006	*	*	*	*	**	*	*	*	9
Saigal et al. [29]	2007	*	*	*	*	*	*	*	*	8
Keating et al. [24]	2010	*	*	*	*	**	*	*	*	9
Van Hemelrijck et al. [31]	2010	*	*	*	*	*	*	*	*	8
Kim et al [25].	2011	*	*	*	*	*	*	*	*	8
Hu et al. [22]	2012	*	*	*	*	*	*	*	*	9
O’Farrell et al. [27]	2015	*	*	*	*	**	*	*	*	9
Teoh et al. [30]	2015	*	*	*	*	**	*	*	*	9
Morgia et al. [26]	2016	*	*	*	*	**	*	*	*	9
Oka et al. [28]	2016	*	*	*	*	*	*	*	*	8
Nguyen et al. [33]	2018	*	*	*	*	*	*	*	*	8
Wu et al. [4]	2020	*	*	*	*	**	*	*	*	9

 Table 4 shows the quality assessment of cross-sectional studies using the modified version of the NOS.

**Table 4:** Modified version of the Newcastle-Ottawa Scale: cross-sectional studies.

Author	Year	Selection (maximum of five stars)				Comparability (maximum of two stars)	Outcome (maximum of three stars)		Total score (maximum of 10 stars)
		Representativeness of the sample	Sample size	Non-respondents	Ascertaining risk	Control for important or additional factors	Assessment of outcomes	Statistical test	
Cleffi et al. [18]	2011	*		*	*	**	**	*	8
Gandaglia et al. [21]	2014	*	*	*	*	**	**	*	9

Selection and Data Extraction

Two reviewers separately obtained data utilizing a predefined data extraction form from the final articles after quality assessment. With the help of a third reviewer, disagreements were addressed via debate or consensus. The following information was obtained: first author’s name, sample size, research features (i.e., year, design, and setting), type of ADT intervention used in the study, cardiovascular events described in every paper, and other non-cardiovascular events described in the study. Extracted data are summarized in Table 5.

**Table 5:** Data extraction table. ADT: androgen deprivation therapy; GnRH: gonadotropin-releasing hormone

Study	Country	Year	Study design	Setting/Context	Sample size	ADT type	Cardiovascular events studied	Non-cardiovascular events studied
Keating et al. [23]	United States	2006	Retrospective cohort	Fee-for-service Medicare enrollees	73,196	GnRH agonist bilateral orchiectomy	Coronary artery disease, myocardial infarction, sudden cardiac death	Diabetes
D’Amico et al. [19]	United States, Canada, Australia, and New Zealand	2007	Randomized clinical trial	Pooled data analysis of randomized studies	1,372	GnRH agonist	Myocardial infarction	None
Saigal et al. [29]	United States	2007	Retrospective cohort	Population-based registry, Surveillance Epidemiology and End Results (SEER) database	22,816	Not specified	Cardiovascular morbidity	None
Efstathiou et al. [20]	United States	2009	Randomized clinical trial	The data used in this analysis were based on the RTOG protocol	1,554	GnRH agonist	Cardiovascular mortality	None
Keating et al. [24]	United States	2010	Retrospective cohort	Veterans Healthcare Administration	37,443	GnRH agonist, orchiectomy, combined androgen blockade, oral anti-androgen	Coronary artery disease, myocardial infarction, sudden cardiac death	Diabetes, stroke
Van Hemelrijck et al. [31]	Sweden	2010	Retrospective cohort	PCBaSe Sweden is based on the National Prostate Cancer Register	76,600	Anti-androgens, orchiectomy, GnRH agonists	Systemic inflammation reaction of cardiovascular disease, arrhythmia, ischemic heart disease	None
Cleffi et al. [18]	Brazil	2011	Cross-sectional study	Not mentioned	79	Not specified	Cardiovascular risk	Hypertension, diabetes, metabolic syndrome, hypertriglyceridemia, central obesity
Kim et al. [25]	Canada	2011	Retrospective cohort	Provincial pharmacy and radiotherapy databases were linked to the provincial cancer registry	5,948	Not specified	Cardiovascular mortality	Other causes of mortality
Hu et al. [22]	United States	2012	Retrospective cohort	The US National Cancer Institute Surveillance Epidemiology and End Results (SEER) Medicare data for analyses, a linkage of population-based tumor registry data that currently covers areas representing 28% of the US population with Medicare administrative data	182,757	GnRH agonist	Peripheral arterial disease, venous thromboembolism	None
Gandaglia et al. [21]	United States	2014	Retrospective cohort	Surveillance, Epidemiology, and End Results (SEER), eight Medicare-linked database	40,474	GnRH agonists, bilateral orchidectomy	Coronary artery disease, myocardial infarction, sudden cardiac death	None
O’Farrell et al. [27]	Sweden	2015	Retrospective cohort	Using data on filled drug prescriptions in Swedish national health care registers	41,362	Anti-androgens, gonadotropin-releasing hormone agonists underwent surgical orchiectomy	Cardiovascular risk	None
Teoh et al. [30]	China	2015	Prospective cohort	All Chinese prostate cancer patients from 2000 to 2009	684	Surgical castration, GnRH agonists	Cardiovascular thrombotic risk, myocardial infarction	Ischemic stroke
Morgia et al. [26]	Italy	2016	Cross-sectional study	Patients from 30 Italian institutes, including nine radiotherapy and 21 urology centers	1,386	GnRH agonist, orchiectomy, combined androgen blockade, oral anti-androgen	Cardiovascular prevalence	Osteoporosis
Oka et al. [28]	Japan	2016	Prospective observational study	Patients were at Toho University Sakura Medical Center	58	Not specified	Arterial stiffness	Hyperlipidemia
Nguyen et al. [33]	United States	2018	Retrospective cohort	National Cancer Institute͛s Surveillance, Epidemiology, and End Results (SEER) Program Medicare-linked database	201,797	Not specified	Coronary artery disease, myocardial infarction	Bone fractures, diabetes, dementia, sexual dysfunction
Wu et al. [4]	Taiwan	2020	Retrospective cohort	Data from patients were retrospectively collected from the Longitudinal Health Insurance Database of Taiwan	10,843	Not specified	Hypertension	None

Results

Literature Search

**Figure 2:**
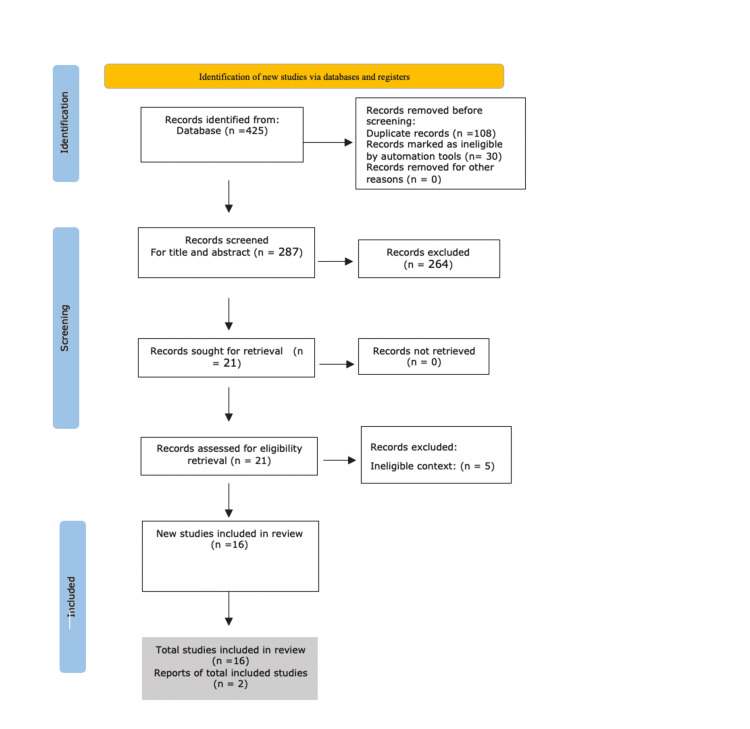
PRISMA flowchart. PRISMA: Preferred Reporting Items for Systematic Reviews and Meta-Analyses

Our extensive search resulted in 425 studies. Only 287 studies remained after removing 108 duplicates. In total, 264 articles were omitted after reviewing the abstracts and titles because they did not fit the inclusion criteria for the following causes: secondary studies, laboratory searches, and non-relevant topics. Finally, the entire paper was carefully read, of which five were further excluded because of non-cardiovascular end-point. Figure 2 shows the PRISMA flowchart demonstrating the search process and study selection.

Study Characteristics

In total, 14 observational studies and two randomized control trials (RCTs ) were included in the systemic review [4,19-33]. The key characteristics of the investigations (published between 2006 and 2020) that were considered are listed in Table 5. Seven studies were performed in America, one in Japan, two in Sweden, one in China, two in Canada, one in Taiwan, and one in Brazil. One clinical trial was done in the United States, Australia, and Canada. The sample sizes in the reviewed clinical investigations ranged from 79 to 201,797 participants. All studies had ADT as the intervention.

Seven of these studies did not mention the specific ADT type used and compared outcomes between patient groups that used ADT to those that did not. The other studies mention the specific type of ADT used and compared the outcomes between patient groups receiving different types of ADT such as GnRH agonists, combined androgen blockade (CAB), surgical castration, and oral anti-androgen with each other and with the patient group that did not receive ADT.

ADT Versus Non-ADT Groups on Cardiovascular Events

Myocardial infarction (MI), sudden cardiac death (SCD), and CAD: According to a study by Wallis et al., radiation (adjusted hazard ratios (aHR) = 1.16-1.28, p < 0.0001-0.04) and ADT (aHR = 1.18-1.32, p < 0.0001-0.0008) were both linked with an elevated risk of CAD, SCD, and fracture requiring hospitalization after adjusting for baseline differences [32]. Radiotherapy was associated with a greater risk of MI (aHR = 1.20, p = 0.02) but not ADT (p = 0.5) [32]. In a study by Nguyen et al., individuals who used ADT had a greater risk of CAD (HR = 1.12, 95% confidence interval (CI) = 1.09-1.14) and acute MI (HR = 1.11, 95% CI = 1.08-1.15) than those who did not [33].

Standardized incidence ratio (SIR) of CVD, arrhythmia, and ischemic heart disease (IHD): Van Hemelrijck et al. reported that regardless of the history of cardiovascular illness, SIR for CVD was high in all males undergoing ADT, with the greatest values among those on endocrine treatment [31]. In this study, endocrine treatment was grouped into anti-androgens, estrogens, orchiectomy, GnRH agonists, GnRH agonist combined with long-term anti-androgens, and other types of endocrine therapy. SIR MI for men without circulatory disease history: 1.40 (95% CI = 1.31 to 1.49), 1.15 (95% CI = 1.01 to 1.31), and 1.20 (95% CI = 1.11 to 1.30) for men undergoing primary endocrine therapy, radical prostatectomy/radiotherapy, and surveillance endocrine therapy, respectively.

Arterial stiffness: Within six months following ADT, the entire sample exhibited no significant increase in arterial stiffness, according to Oka et al., although 55.2% of patients had an elevated cardio-ankle vascular index (CAVI) [28]. Total cholesterol, high-density lipoprotein cholesterol (HDL-C), and low-density lipoprotein cholesterol (LDL-C) levels in the blood significantly increased one month after starting ADT and remained high thereafter.

Hypertension: Over a five-year follow-up period, Wu et al. found that the incidence of new-onset hypertension was 22.6 per 1,000 person-years in patients who did not receive ADT and 33.0 per 1,000 person-years in those who did receive ADT [4]. There was a 1.78-fold enhanced risk of developing new-onset hypertension in the group that received ADT than in the control group (95% CI = 1.61-1.96; p < 0.001). Furthermore, the CAB group had almost doubled the likelihood of subsequent hypertension (aHR = 1.93; 95% CI = 1.71-2.18; p < 0.001).

Cardiovascular morbidity: According to Saigal et al., patients with prostate cancer who had ADT for at least one year had a 20% greater risk of substantial cardiovascular morbidity than those who did not [29]. The individuals began to encounter this elevated risk after just 12 months of treatment.

Cardiovascular mortality: According to Kim et al., patients with no ADT, six months of ADT, and more than six months of ADT, respectively, had cumulative cardiovascular mortality of 2.6% (95% CI = 1.9-3.5%), 2.1% (95% CI = 1.2-3.5%), and 1.4% (95% CI = 1.0-2.0%) at seven years (Gray’s p = 0.002) [25]. In this study, compared to the more than six-month ADT group, the non-ADT group had greater cardiovascular disease and risk factors at the start.

GnRH Agonist Versus Surgical Castration Versus CAB Versus Anti-androgens on Cardiovascular Events

General risk of CVD: According to a study by O'Farrell et al., males who used GnRH agonists (HR = 1.21, 95% CI = 1.18-1.25) had a greater risk of CVD than the orchiectomy group (HR = 1.16; 95% CI = 1.08-1.25) and that of the anti-androgen group (HR = 0.87; 95% CI = 0.82-0.91) [27]. CVD risk was higher in males who had two or more cardiovascular incidents before starting ADT, with HR of CVD with GnRH agonist treatment of 1.91 (95% CI = 1.66-2.20), HR of CVD with anti-androgen therapy of 1.60 (95% CI = 1.24-2.06), and HR of CVD with orchiectomy of 1.79 (95% CI = 1.16-2.76) versus comparison cohort. Teoh et al., according to the Kaplan-Meier analysis, determined that the orchiectomy group had a higher incidence of new cardiovascular thrombotic events than the GnRH agonist group (p = 0.014) [30]. Age (HR = 1.072, 95% CI = 1.04-1.11; p = 0.001), hyperlipidemia (HR = 2.455, 95% CI = 1.53-3.93; p = 0.001), and orchiectomy (HR = 1.648, 95% CI = 1.05-2.59; p = 0.031) were all found to be significant risk factors for cardiovascular thrombotic events according to multivariate Cox regression analysis.

CVD prevalence: In a study by Morgia et al., a total of 1,075 people were included, with 285 (26.51%) and 790 (73.49%) being discordant and concordant, respectively, according to the EAU criteria [26]. Discordant ADT was linked to a higher incidence of cardiovascular issues (odds ratio (OR) = 2.07; p = 0.01) in a multivariate logistic regression analysis adjusted for confounding factors, with GnRH agonists (HR = 3.95, 95% CI = 1.01-15.34; p = 0.005) and CAB (HR = 3.37, 95% CI = 1.10-10.30; p = 0.005) both contributing to cardiovascular complications.

CAD, MI, and SCD: According to Keating et al., treatment with GnRH agonists was linked to statistically significantly greater risks of incident CAD (aHR = 1.19, 95% CI = 1.10-1.28), MI (aHR = 1.28, 95% CI = 1.08-1.5) [23,24]. Oral anti-androgen monotherapy was not linked to any of the outcomes investigated. According to Gandaglia et al., overall, the rates of CAD, MI, and SCD were 25.9%, 15.6%, and 15.8%, respectively after 10 years [21]. After stratification by ADT status (ADT-naive vs. GnRH agonists vs. bilateral orchidectomy), the CAD rates were 25.1%, 26.9%, and 23.2%, respectively. The acute MI rates were 14.8%, 16.6%, and 14.8%, while the SCD rates were 14.2%, 17.7%, and 16.4%, respectively. GnRH agonists (all p = 0.001) but not bilateral orchidectomy (all p = 0.7) were linked to a greater risk of CAD, MI, and SCD in competing-risk multivariable regression models. According to Amico et al., males aged 65 and over who got six months of ADT had shorter delays to fatal MIs than men in this age group who did not get ADT (p = 0.017) or men younger than 65 years (p = 0.016) [19]. The time to fatal MIs in males aged 65 and older who got six to eight months of ADT compared to three months of ADT revealed no significant difference (p = 0.97). Here, ADT used was GnRH agonists.

Peripheral arterial disease (PAD) and venous thromboembolism (VTE): GnRH agonist usage was linked to a greater risk of PAD (aHR = 1.16; 95% CI = 1.12-1.21) and VTE (aHR = 1.10; 95% = CI 1.04-1.15). Orchiectomy was also linked to an elevated risk of PAD (aHR = 1.13; 95% CI = 1.02-1.26) and VTE (aHR = 1.27; 95% CI = 1.11-1.45), according to Hu and colleagues [22].

Cardiovascular mortality: According to Efstathiou et al., cardiovascular mortality was 5.9% for men getting longer-term adjuvant goserelin versus 4.8% for men receiving short-term goserelin after five years (Gray’s p = 0.16). In multivariate analyses, the treatment arm was not linked to an enhanced risk of cardiovascular mortality when censoring at the time of salvage goserelin (aHR = 1.02, 95% CI = 0.73-1.43; p = 0.9) [20]. Traditional cardiac risk variables such as age, CVD, and DM were all linked to a greater risk of CVD [20].

Discussion

Systematic reviews are considered the highest level of evidence and are typically emphasized in evidence-based practice because they reduce the errors and biases that can be introduced by single research. A well-conducted systematic review can assist researchers in objectively establishing the boundaries of what is known and what is unknown by summarizing all of the relevant and reliable evidence to enhance clinical decision-making [34,35]. This review attempted to involve all potentially relevant literature related to the research topic, which was cardiovascular events due to ADT in prostate cancer patients.

ADT may be beneficial to prostate cancer patients as part of curative treatment or as a palliative treatment for advanced disease. As the average life expectancy for males with prostate cancer increases, more people may be exposed to the possible adverse effects of ADT over longer periods. Care for cardiovascular events is an important element of the survivorship phase for a significant number of prostate cancer patients.

The 16 articles considered in the review were good quality studies as per the NOS and Cochrane risk bias assessment tools, out of which 14 were observational studies and two RCTs. Seven of these studies did not mention the specific ADT type used and compared outcomes between patient groups that use ADT to those that did not. The other studies mentioned the specific type of ADT used and compared the outcomes between patient groups receiving different types of ADT such as GnRH agonists, CAB, surgical castration, and oral anti-androgen with each other and with the patient group that did not receive ADT.

ADT Versus Non-ADT Groups on Cardiovascular Events

According to Wallis et al., there is an increase in CAD and SCD in both the groups individually but there is an increase in MI only in the non-ADT group [32]. When discussing the risks and benefits of treatment for localized prostate cancer for formulating a survivorship plan, the increased use of ADT for males with localized disease undergoing radiotherapy, and the observed higher prevalence of CAD, MI, and SCD in these patients should be considered. Nguyen et al. conducted a study that found males who received ADT had a higher incidence of CAD, MI, and SCD who did not receive ADT [33].

According to Van Hemelrijck et al., all prostate cancer patients, especially those treated with ADT, have enhanced relative risks of fatal and non-fatal CVD (SIR of CVD, arrhythmia, IHD) [31]. According to Oka et al., while the entire cohort did not show a significant change in arterial stiffness with ADT, some patients with CAVI showed an increase in arterial stiffness [28]. The ratio of LDL-C to HDL-C, or LDL-C/HDL-C may impact the development of arterial stiffness following ADT administration. Thus, doctors may be able to use LDL-C/HDL-C values to monitor prostate cancer patients who are at high risk of developing arterial stiffness following ADT therapy in prostate cancer patients.

According to the findings of Wu et al., males who had ADT for prostate cancer are at risk of having hypertension in the future [4]. According to Saigal et al., ADT is related to a significantly higher cardiovascular morbidity in prostate cancer patients and may reduce overall survival in low-risk men [29]. These findings are especially important for deciding whether or not to use ADT in prostate cancer patients in situations where the benefit has not been proven. According to Kim et al., individuals who received longer durations of ADT had a lower cardiovascular mortality rate than those who did not get ADT [25]. These discrepancies are most likely because of patient selection for ADT rather than the impact of ADT.

GnRH Agonist Versus Surgical Castration Versus CAB Versus Anti-androgens on Cardiovascular Events

According to Morgia et al., almost one-third of prostate cancer patients got inappropriate GnRH agonists who in turn had a higher prevalence of CVD [27]. Cardiovascular risk is significantly increased in males who received ADT in the form of GnRH agonists and surgical castration. However, prostate cancer patients who took anti-androgen had a lower chance of developing CVD risk factors. According to the findings of O'Farrell et al., there should be a strong indication for ADT in males with prostate cancer such that the benefits outweigh the risks; this is especially important in males with a recent history of CVD [28]. When compared to GnRH agonists, Teoh et al. claim that surgical castration is linked to a greater risk of cardiovascular thrombotic events. This is an essential factor to consider when choosing an ADT approach, particularly in older men with a history of hyperlipidemia [30].

GnRH agonist treatment for males with locoregional prostate cancer was linked to an elevated risk of diabetes and cardiovascular disease (CAD, MI, and SCD), according to Keating et al. (2006) [23]. ADT with GnRH agonists was linked to an elevated risk of diabetes and CVD (CAD, MI, and SCD), ADT with CAB was linked to an enhanced risk of CAD, ADT with orchiectomy was linked to CAD and MI, and oral anti-androgen monotherapy was not linked to any of the outcomes studied, according to Keating (2010) [24]. In males with non-metastatic prostate cancer, Gandaglia et al. revealed that the administration of GnRH agonists, but not orchidectomy, is still associated with a considerably higher risk of CAD, MI, and, especially SCD. In men with a higher risk of CVD, alternative types of ADT should be evaluated [22]. Amico et al. discovered that GnRH agonist use is linked to an earlier onset of fatal MIs in males 65 and older who are treated for six months versus males who are not treated with GnRH agonist [19].

Hu et al. state that ADT in the forms of GnRH agonists and surgical castration for non-metastatic prostate cancer is linked to an enhanced risk of PAD and VTE. The observational research design has limitations as does the inability to examine the usage of oral anti-androgens [22]. According to Efstathiou et al., a longer duration of adjuvant GnRH agonist medication does not improve cardiovascular mortality in males with locally advanced prostate cancer, whereas traditional cardiac risk factors, such as DM, prevalent CVD, and age, were significantly associated with higher cardiovascular mortality [20].

## Conclusions

Although ADT has several negative metabolic side effects that increase the likelihood of cardiovascular toxicity, published studies utilizing various designs have discovered inconsistencies in the impact of ADT on cardiovascular outcomes. In those who use ADT, the incidence of cardiovascular events such as MI, SCD, CAD, hypertension, and overall cardiovascular morbidity is higher than in those who do not. GnRH agonists, when compared to other types of ADT, have a higher incidence of cardiovascular events such as MI, SCD, CAD, PAD, and VTE. ADT, on the other hand, does not increase cardiovascular mortality in men with locally advanced prostate cancer. The findings suggest that while CVD risk should be considered when prescribing ADT, it must not be considered a contraindication if the expected benefit is significant.* *Systematic reviews can help, but they can never take the place of excellent clinical reasoning. Availability of time and resources, experience, and compliance should all be considered when tailoring treatment to unique individuals and situations.
